# Comparative Genomic Characterization of *Riemerella anatipestifer* Isolates from Chickens, Ducks, and Geese in China

**DOI:** 10.3390/ani16182847

**Published:** 2026-09-10

**Authors:** Yunqing Guo, Rongrong Zhang, Tengfei Zhang, Wenting Zhang, Qin Lu, Qiao Hu, Qingping Luo

**Affiliations:** 1 Key Laboratory of Prevention and Control Agents for Animal Bacteriosis, Ministry of Agriculture and Rural Affairs, Institute of Animal Sciences and Veterinary Medicine, Hubei Academy of Agricultural Sciences, Wuhan 430064, China; 2 Key Laboratory of Animal Pathogenic Microbiology of Hubei Province, Institute of Animal Sciences and Veterinary Medicine, Hubei Academy of Agricultural Sciences, Wuhan 430064, China; 3 Hubei Hongshan Laboratory, Wuhan 430070, China

**Keywords:** *Riemerella anatipestifer*, comparative genomics, host-associated genomic differences, China

## Abstract

The increasing detection of *Riemerella anatipestifer* in chickens poses severe challenges to the poultry industry. However, the genomic differences among *R. anatipestifer* isolates from different poultry hosts remain poorly understood. Here, we investigated host-associated genomic differences among 158 *R. anatipestifer* strains isolated from different hosts in China, using pan-genome, phylogenetic, virulence factor, and antimicrobial resistance analyses. The core-genome phylogeny showed no apparent separation according to host, sampling period, or geographic origin. Conserved genes including *groEL*, *tufA*, and *katB* were detected in all strains, whereas *tviB* and *ureG* displayed host-associated distribution patterns. More than 40 putative antimicrobial resistance (AMR) genes were detected across 158 *R. anatipestifer* strains, indicating the presence of multiple putative AMR determinants. Among these, *bla_RAD-1_*, *bla_RASA-1_*, and *dfrA36* exhibited host-associated distribution patterns. Pan-genome analysis further identified host-associated genomic differences in *R. anatipestifer*. These included genes contributing to cell-surface modification pathways mediated by *pglH*, and genes involved in toxin–antitoxin system-dependent stress regulation via *parD1/parE1* and *yefM-yoeB*. Overall, our comparative genomic characterization of *R. anatipestifer* characterizes host-associated genomic differences and provides a basis for further investigation of their biological significance.

## Simple Summary

*Riemerella anatipestifer* causes severe contagious disease in waterfowl and has increasingly been detected in chickens, threatening sustainable poultry production. To investigate host-associated genomic differences, we performed comparative genomic analyses of 158 Chinese field isolates from chicken, duck and goose. The core-genome phylogeny showed no apparent separation according to host, sampling period, or geographic origin. However, virulence and antimicrobial resistance genes exhibited host-associated distribution patterns. Pan-genome analysis identified host-associated differences in genes involved in cell-surface modification and stress responses. Overall, this comparative analysis of chicken-, duck-, and goose-derived isolates provides insight into host-associated genomic differences in *R. anatipestifer* and may inform future research on disease prevention and control.

## 1. Introduction

*Riemerella anatipestifer* is a Gram-negative, non-motile, rod-shaped bacterium belonging to the family *Flavobacteriaceae*. The primary hosts of *R. anatipestifer* are waterfowl, including ducks and geese, as well as turkeys. Although chickens are considered less susceptible to infection, increasing outbreaks of chicken-derived *R. anatipestifer* infection have been reported in major poultry-producing regions, including China, Greece, and Poland [1,2].

In waterfowl, *R. anatipestifer* infection primarily causes septicaemia and infectious serositis and can result in substantial economic losses owing to high incidence and mortality rates among young birds [3]. Chickens infected with *R. anatipestifer* exhibit clinical signs similar to those seen in infected waterfowl. Furthermore, infection can markedly reduce egg production and hatchability. In chickens, infection has been associated with “jelly-like embryos” and fibrinous lesions such as airsacculitis, arthritis, and oviduct obstruction [4]. Multiple independent duck-to-chicken spillover events have also been identified through ancestral state reconstruction [5].

Despite the increasing detection of chicken-derived *R. anatipestifer*, the genomic differences among isolates from different poultry hosts remain incompletely characterized. Moreover, *R. anatipestifer* genomes have been reported to harbor numerous putative antimicrobial resistance (AMR) determinants. Therefore, we aimed to characterize host-associated genomic differences in gene content, putative virulence factors, and AMR determinants among *R. anatipestifer* isolates from different hosts and time periods. The findings provide genomic data that may facilitate further investigation of the epidemiology and control of *R. anatipestifer*.

## 2. Materials and Methods

### 2.1. Bacterial Strains

The genome sequences of 158 *R. anatipestifer* strains were analyzed, including 40 strains isolated in this study and 118 genomic sequences downloaded from the NCBI database. The 40 strains isolated in this study were obtained from independent farms with diseased commercial broilers and layer farms across Shandong, Hubei, and Liaoning provinces. The 118 *R. anatipestifer* strains were randomly sampled from all Chinese *R. anatipestifer* isolates available in NCBI from 2011 to 2025. For isolates from the same region and sampling year, a single strain was randomly selected to avoid duplication, while maintaining coverage of all the available sampling years, regions, and host species. Detailed information on the strains is provided in Table S1.

### 2.2. Strain Isolation and Genome Sequencing

Anal swabs were collected, streaked on tryptic soy agar (TSA) supplemented with 5% newborn calf serum and incubated at 37 °C in 5% CO_2_ for 48 h. Bacterial cultures were prepared by inoculating single colonies into tryptic soy broth (TSB) supplemented with 5% calf serum and incubating them at 37 °C with shaking at 180 rpm overnight. *R. anatipestifer* identity was confirmed using 16S rDNA PCR (RA-5: 5′-TCGAGATTGCATCACTTCGCATTG; RA-3: 5′-GCTAGTCTTGAGTATAGTTGA GCT AGC-3′) [2]. PCR was performed using primers with an annealing temperature of 50 °C, yielding a 645 bp amplicon.

Genomic DNA was extracted using a Bacterial DNA Kit (Tiangen, Beijing, China) according to the manufacturer’s instructions. Genome sequencing was performed by Shanghai Majorbio Bio-Pharm Technology Co., Ltd. (Shanghai, China). Genomic DNA was fragmented to approximately 400 bp using a Covaris M220 Focused-Acoustic Shearer (Covaris, Woburn, MA, USA). DNA libraries for Illumina paired-end sequencing were constructed from the fragmented DNA utilizing the NEXTFLEX Rapid DNA-Seq Kit (Revvity, Waltham, MA, USA). The final libraries were sequenced using 2 × 150 bp paired-end sequencing on an Illumina NovaSeq 6000 platform (Illumina Inc., San Diego, CA, USA). Raw sequencing reads were preprocessed and quality-filtered using fastp version 0.19.6 (https://github.com/OpenGene/fastp, accessed on 25 August 2026). Raw reads were processed as follows: trim read termini with base Phred quality scores below Q20, filter out reads containing >10% ambiguous N-bases, and discard fragments shorter than 25 bp after adapter clipping and quality trimming. The filtered reads were subsequently assembled de novo using SPAdes version 3.15.5 (http://cab.spbu.ru/software/spades/, accessed on 25 August 2026). Genome coverage calculated based on read mapping reached 100%. The genome coverage of the isolates, calculated via the read-mapping approach, reached 100%, demonstrating full capture of the target genomic sequences.

### 2.3. Phylogenetic Analysis

Coding sequences were uniformly predicted using Prokka (version 1.15.6) with the--noanno, --norrna, and --notrna options, generating standardized protein-coding sequences and GFF/FAA files. The pan-genome was inferred using Panaroo (version 1.6.0) with strict cleaning, gene refinding disabled (--refind-mode off), and a core-gene threshold of 95%. Core genes were aligned using MAFFT (version 7.526). Recombinant regions were identified and masked using Gubbins (version 3.4.3). RAxML-NG (version 2.0.2) was used for construct maximum-likelihood tree construction during Gubbins iterations, with a maximum of five iterations. The final maximum-likelihood phylogeny was inferred using IQ-TREE (version 2.4.0) with ModelFinder and the MFP + ASC model-selection strategy. Branch support was assessed using 1000 ultrafast bootstrap replicates and 1000 SH-aLRT replicates. The best-fitting model for the final tree was TVM + F + ASC + R2.

### 2.4. Pan-Genome Analysis

The annotated genomes were used for pan-genome analysis to characterize the core (orthogroups present in ≥95% of isolates), accessory (orthogroups present in <95% but ≥1 of isolates) and host-associated genes (FDR/q-value < 0.05 and odds ratios (OR) > 1). A sequence similarity threshold of 95% was used to identify homologous gene clusters among strains. Specifically, for each orthogroup, we compared the gene prevalence within one host group against that across the remaining host cohorts, with the total strain count of each host category set as the background population to calculate corresponding *p*-values. Fisher’s exact test was used to identify host-associated genes (Table S2). The *p*-values were corrected using the Benjamini–Hochberg false discovery rate (FDR) method (Table S3). Genes with *p* < 0.05 and FDR/q-value < 0.05 were considered significantly enriched in the corresponding host.

### 2.5. Virulence Factor Analysis

ABRicate v1.0.1 was used to screen the whole-genome sequences of all strains against the Virulence Factor Database (VFDB; updated April 2025) for virulence factor analysis (Table S4). Alignment hits for virulence factor were filtered using thresholds of ≥60% identity and ≥60% coverage.

### 2.6. AMR Gene Analysis

ABRicate was used to align the whole-genome sequences of all strains against the Comprehensive Antibiotic Resistance Database (CARD; updated April 2025) for AMR gene analysis. Alignment hits for AMR genes were filtered using thresholds of ≥60% identity and ≥60% coverage. ResFinder was used to validate mobile resistance genes. Only resistance determinants using the integrated CARD and ResFinder screening strategy are reported (Table S5).

## 3. Results and Discussion

In this study, 97 duck-derived, 28 chicken-derived, and 33 goose-derived isolates were included. The bacterial pan-genome comprises core and dispensable genomes, representing the conserved and variable components of the gene repertoire, respectively. The analysis identified 5412 gene clusters, including 1469 core-gene clusters, yielding a concatenated core-gene alignment of 1,527,901 bp. Of these, 1423 were present in 99–100% of genomes and 46 in 95–<99%.

### 3.1. Core-Genome Phylogenetic Analysis

**Figure 1:**
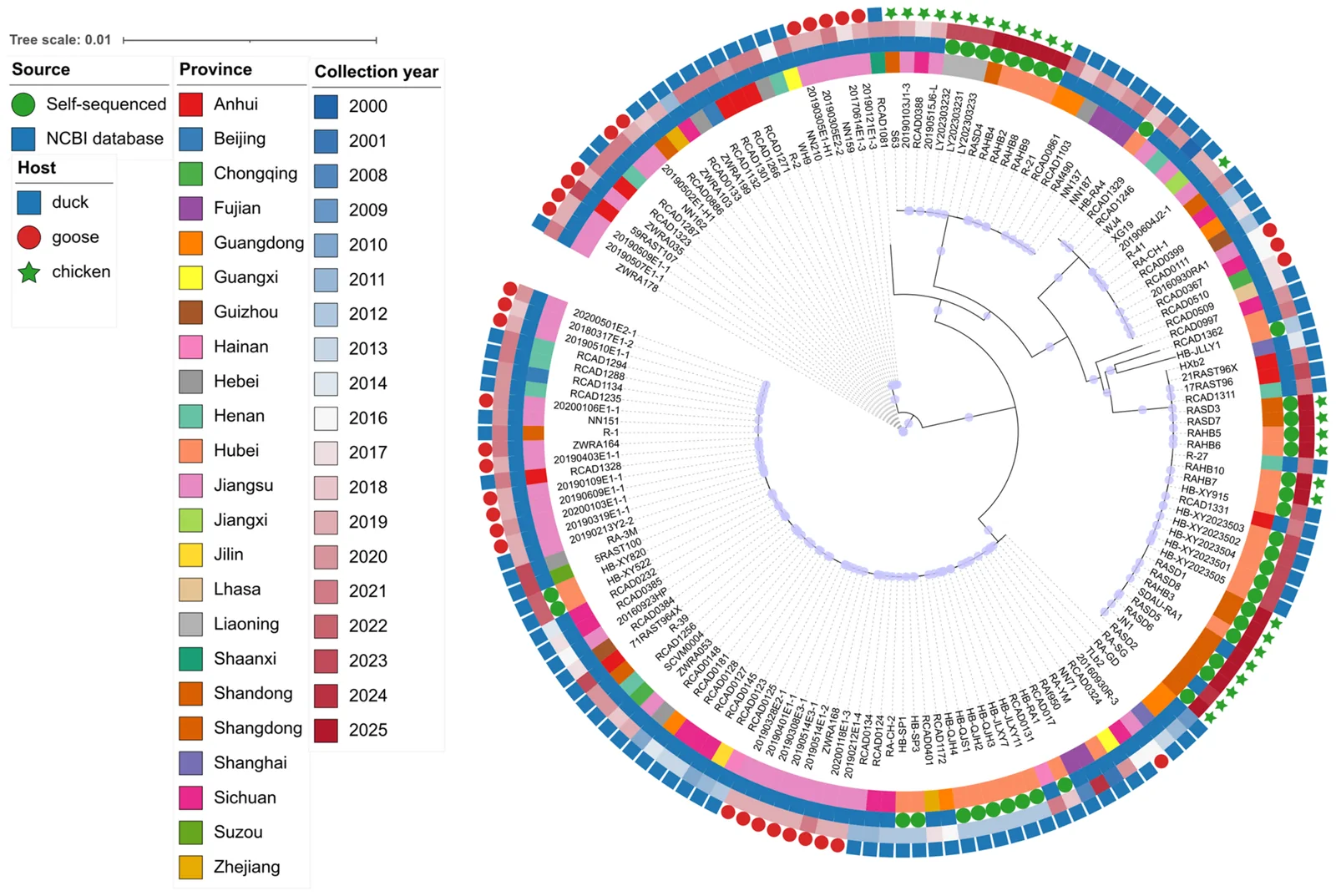
Core-genome phylogenetic tree of *R. anatipestifer* strains from different hosts, time periods, and provinces in China.

The core-genome phylogenetic tree was constructed using single-copy orthologs after recombination filtering. The core-genome phylogeny showed no apparent separation according to host, sampling period, or geographic origin (Figure 1). This finding indicates that there is no clear host-associated phylogenetic separation among the analyzed *R. anatipestifer.* This is consistent with the findings of previous genomic studies that showed no strict host-associated phylogenetic separation [6].

### 3.2. Pan-Genome Analysis

**Figure 2:**
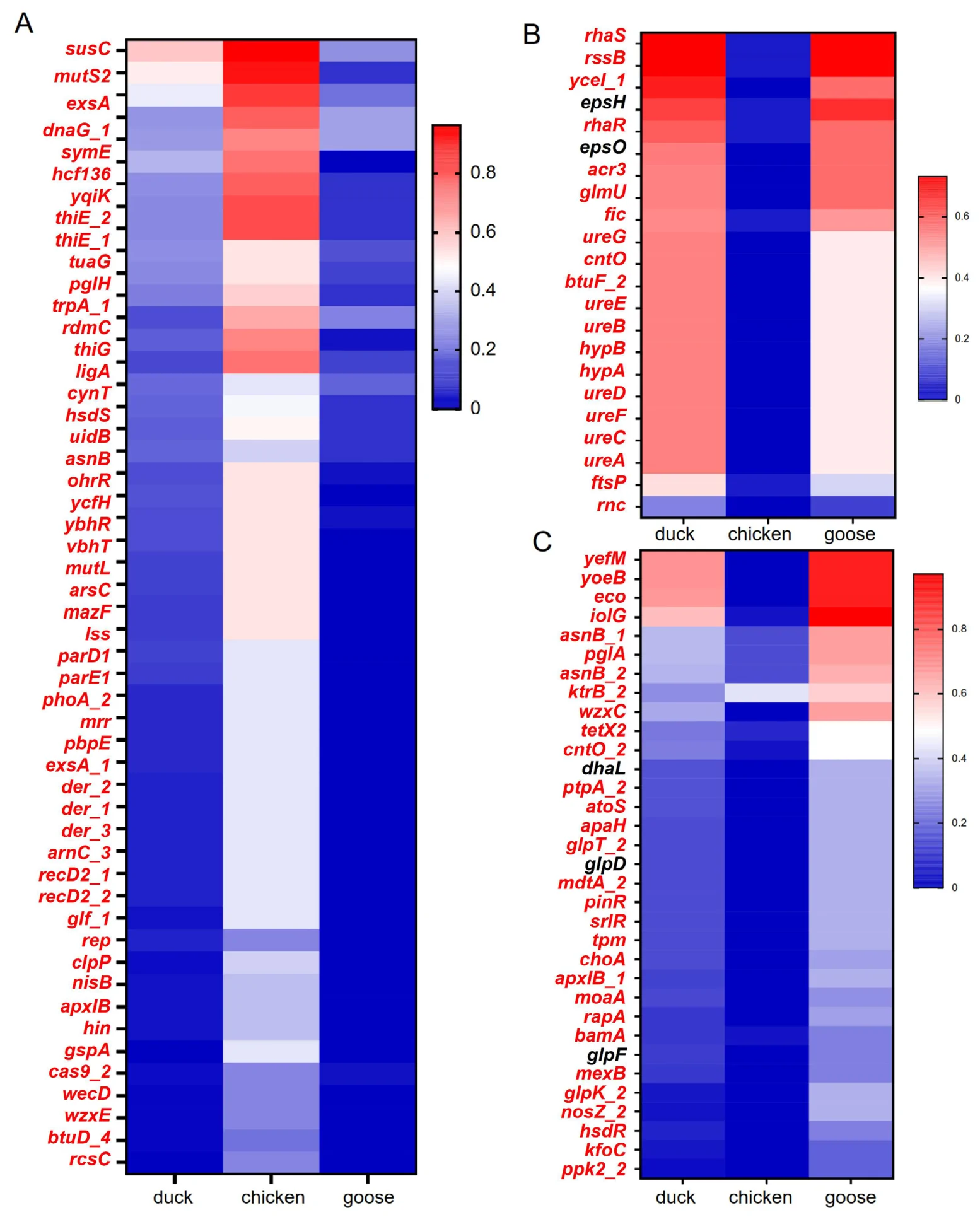
Analysis of host-associated genes of *R. anatipestifer* strains from different hosts. (**A**) Host-associated in chicken-derived strains. (**B**) Host-associated in duck-derived strains. (**C**) Host-associated in goose-derived strains. Genes shown in red exhibit significant differences after FDR correction (*p* < 0.05 and FDR/q-value < 0.05). Genes shown in black are not significantly different by FDR criteria (*p* < 0.05 but FDR/q-value > 0.05).

Pan-genome analyses revealed several host-associated gene clusters (Figure 2). Cell surface biosynthesis plays a critical role in host adaptation and immune evasion by modulating surface antigenicity, charge, and immune recognition. In chicken-derived strains, the enriched genes included *pglH* (involved in protein glycosylation), *tuaG* (glycosyltransferase within the teichuronic acid biosynthetic operon), and *wecD*/*wzxE* (polysaccharide biosynthesis-related genes). Genes associated with stress/virulence regulation (*ohrR*, *mazF*, *symE*, *parD1*/*parE1*) were also enriched in chicken-derived strains. *mazF*, *parD1*/*parE1*, and *symE* belong to the toxin-antitoxin (TA) systems, which have been associated with prokaryotic stress tolerance and environmental responses. Notably, the *yefM–yoeB* system was absent from chicken-derived *R. anatipestifer* in this study. However, it was enriched in the goose-derived isolates. Nevertheless, owing to the limited number and low genetic diversity of chicken-derived isolates, larger sample sets and targeted PCR assays are needed to further validate the absence of the *yefM-yoeB* system in chicken-derived strains.

Chicken-derived strains displayed significant enrichment of genes involved in DNA repair and recombination (*mutS2*, *mutL*) and signal transduction (*rcsC*) (Figure 2A). The *mutS* and *mutL* genes encode core components of the bacterial mismatch repair pathway, which mediates genomic genetic variation and contributes to the maintenance of genomic stability. Metabolism-related genes were enriched in duck-derived strains (Figure 2B). Host-associated genes enriched in duck-derived strains included a complete urease cluster (*ureA–ureG*) and cofactor assembly factors (*hypA* and *hypB*), which are associated with nitrogen metabolism. Functionally, this intact urease system that hydrolyzes urea can promote bacterial survival on host mucosal surfaces, aligning well with the identified host-associated virulence profiles. Meanwhile, duck isolates showed enrichment of zinc uptake genes and the vitamin B12 transporter gene *btuF_2*, which are associated with metal and nutrient acquisition. Genes related to metabolic and carbon utilization genes were enriched in the goose-derived strains (Figure 2C). These included myo-inositol 2-dehydrogenase encoding gene *iolG*, and 6-phosphogluconolactonase encoding gene *pglA*.

Thus, DNA repair systems, nutrient acquisition pathway, and TA system-mediated stress regulatory networks showed host-associated distribution patterns. These host-associated genes warrant functional validation to determine their biological significance in *R. anatipestifer*.

### 3.3. Virulence Factor Analysis

**Figure 3:**
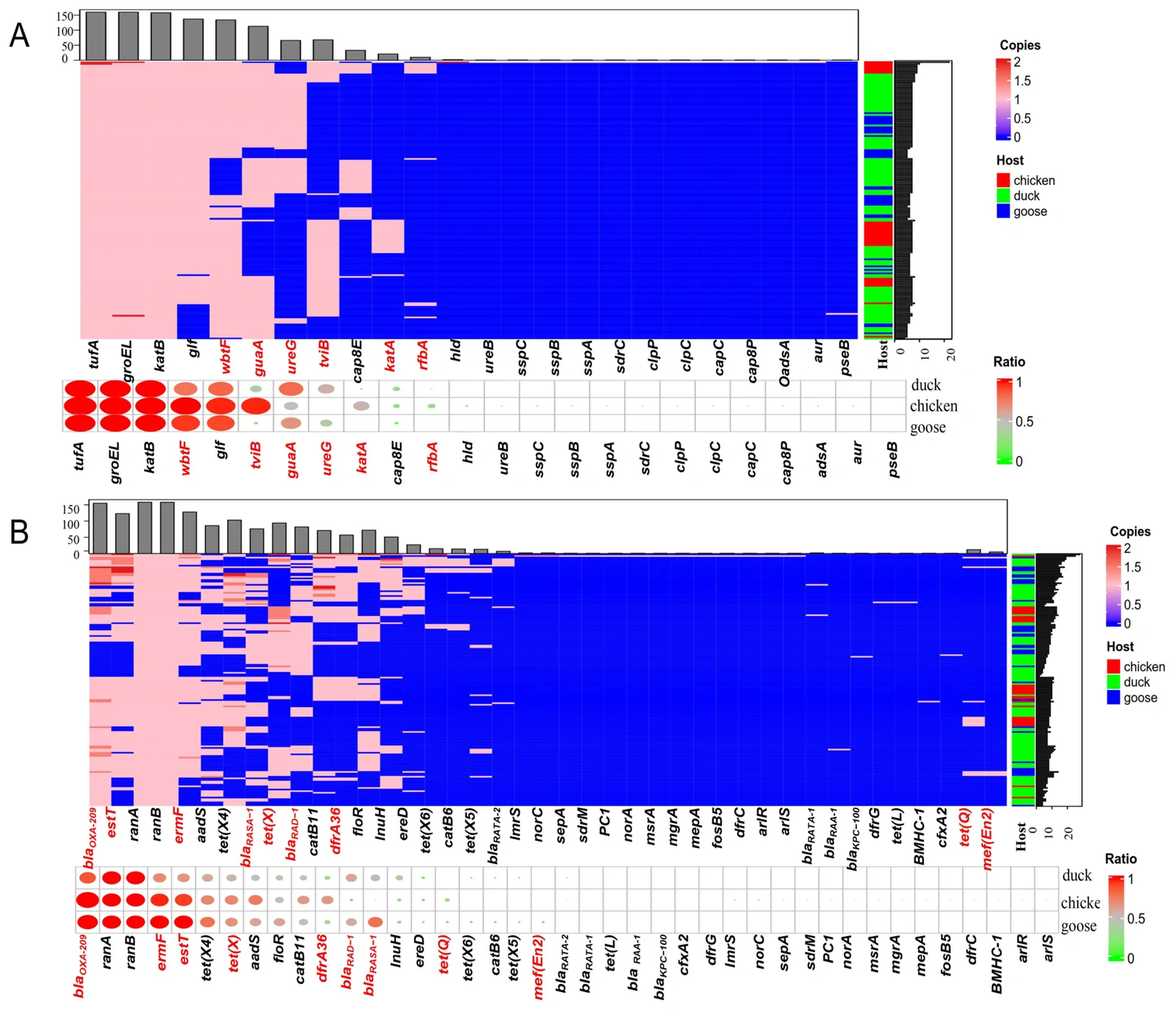
Analysis of virulence factors, AMR genes in *R. anatipestifer* strains from different hosts. (**A**) Virulence factors. (**B**) AMR genes. The heatmap shows the prevalence of genes among all isolates. The bubble chart shows the distribution of genes among different hosts. Genes shown in red exhibit significant differences after FDR correction (*p* < 0.05 and FDR/q-value < 0.05). Genes shown in black are not significantly different by FDR criteria (*p* < 0.05 but FDR/q-value > 0.05).

Among all strains, 24 putative virulence genes were annotated using VFDB, with *groEL*, *tufA*, and *katB* being the most common (Figure 3A). The *groEL*, *tufA*, and *katB* were detected in all strains. *groEL* is involved in the response to environmental stress. *tufA* contributes to bacterial adhesion, thereby affecting cell invasion and dissemination. *katB* is associated with protection against peroxide stress. *glf* and *wbtF* were present in more than 80% of genomes. These findings indicated that *groEL*, *tufA*, and *katB* are conserved genes in *R. anatipestifer*. Their conservation across the analyzed isolates warrant further evaluation as potential vaccine-antigen candidates.

Several low-frequency putative virulence factors were also identified, including type 8 capsular polysaccharide-related genes *cap8E*/*cap8P*/*capC*, *rfbA*, *pseB*, and *sdrC* and T9SS-related genes *sspA*/*sspB*/*sspC*. Low-frequency virulence factors may be involved in surface-antigen modification and interactions with host immune responses [7].

Variable putative virulence factors (*tviB*, *ureG*, and *rfbA*) were detected in a subset of strains, showing host-associated differences in genomic distribution. Previous studies have shown that alterations in capsule structure in *R. anatipestifer* significantly impair its survival in the blood, resistance to complement-mediated killing, and pathogenicity [8]. *tviB*, encoding capsular polysaccharide, was predominantly detected from chicken-derived strains, whereas *ureG*, part of the urease cluster, was enriched in duck-derived strains and absent in chicken-derived strains. This is consistent with the result of pan-genome host-association analysis.

### 3.4. AMR Gene Analysis

The results showed over 40 putative AMR genes associated with transport systems, multidrug efflux pumps and acquired resistance (Figure 3B). Among the highly prevalent resistance genes, *ranA* and *ranB* (predicted ABC efflux pump-encoding genes) were detected in all strains; the β-lactamase-related genes *bla_OXA-209_*, *bla_RAD-1_*, *bla_RASA-1_* and *tet(X)* were detected in 155, 82, 76 and 94 strains, respectively. This indicates that *R. anatipestifer* possesses genomic potential for tetracycline- and tigecycline-resistance. In addition, *bla_OXA_-like* and *tet(X)* have been shown to exhibit high prevalence in *R. anatipestifer* in previous AMR gene analyses [9]. *ErmF*, associated with macrolide/lincosamide resistance, was detected in 128 strains, and *floR*, related to chloramphenicol/florfenicol resistance, was detected in 72 strains.

CARD annotation also revealed host-associated differences in the distribution of AMR determinants. The carbapenem-hydrolyzing class D β-lactamase *bla_RAD-1_* and class A extended-spectrum β-lactamase *bla_RASA-1_* were significantly less frequent in chicken-derived strains than in duck- and goose-derived strains [10,11]. Conversely, the dihydrofolate reductase gene *dfrA36* was more common in chicken-derived strains than in waterfowl-derived strains (Figure 3B). Differences in antimicrobial use among production systems may have contributed to these patterns, but this possibility cannot be distinguished in the present study from temporal, geographic, or lineage effects. Furthermore, these host-associated AMR determinants require further functional investigation.

This study has several notable limitations. Differences in research attention to *R. anatipestifer* and the regional distribution of waterfowl farming resulted in uneven host sample sizes, geographical heterogeneity, and inconsistent sampling years which may introduce temporal and spatial biases. Moreover, the 118 strain genomes were downloaded from the NCBI database for which antimicrobial exposure, lineage and farm-level confounding factors were unavailable. Accordingly, the present comparative genomic analysis cannot determine the mode of transmission through comparative genomics. Phylogenetic correction for pan-genome-wide association analysis and assessment of phylogenetic robustness will be needed in future studies to reduce confounding phylogenetic effects. Finally, our conclusions are limited to host-associated genomic differences. Further experimental validation is essential to determine the biological functions of these host-associated genes.

## 4. Conclusions

Genomic analysis of 158 *R. anatipestifer* isolates revealed no clear host-associated phylogenetic separation. Core putative virulence genes were widely conserved, while several putative virulence factors displayed host-associated distribution patterns. The genomes of these isolates harbored multiple putative AMR determinants. Genes involved in cell surface remodeling, TA system-regulated stress response and DNA repair capacity were enriched in chicken-derived isolates, whereas waterfowl-derived isolates showed enrichment of genes associated with nutrient utilization. These host-associated genes represent candidate genomic features for further investigation.

## Data Availability

The data presented in this study are available in BioProject at www.ncbi.nlm.nih.gov/bioproject, accessed on 25 August 2026; accession number PRJNA1366646.

## Supplementary Materials

The following supporting information can be downloaded at https://www.mdpi.com/article/10.3390/ani16182847/s1: Excel File Table S1: The genome used in this study; Table S2: pan-genome_host_association_FDR_full; Table S3: pan-genome_host_association_FDR_significant; Table S4: VF_host_comparison_stats; Table S5: AMR_host_comparison_stats.

## Abbreviations

The following abbreviations are used in this manuscript:

AMR	antimicrobial resistance
TSA	tryptic soy agar
TSB	tryptic soy broth
FDR	false discovery rate
OR	odds ratios
CARD	Antibiotic Resistance Database
VFDB	Virulence Factor Database
